# Metal Complexes of Quinolone Antibiotics and Their Applications: An Update 

**DOI:** 10.3390/molecules180911153

**Published:** 2013-09-11

**Authors:** Valentina Uivarosi

**Affiliations:** Department of General and Inorganic Chemistry, Faculty of Pharmacy, Carol Davila University of Medicine and Pharmacy, 6 Traian Vuia St, Bucharest 020956, Romania; E-Mail: uivarosi.valentina@umf.ro; Tel.: +4-021-318-0742; Fax: +4-021-318-0750

**Keywords:** quinolones, metal complexes, applications

## Abstract

Quinolones are synthetic broad-spectrum antibiotics with good oral absorption and excellent bioavailability. Due to the chemical functions found on their nucleus (a carboxylic acid function at the 3-position, and in most cases a basic piperazinyl ring (or another N-heterocycle) at the 7-position, and a carbonyl oxygen atom at the 4-position) quinolones bind metal ions forming complexes in which they can act as bidentate, as unidentate and as bridging ligand, respectively. In the polymeric complexes in solid state, multiple modes of coordination are simultaneously possible. In strongly acidic conditions, quinolone molecules possessing a basic side nucleus are protonated and appear as cations in the ionic complexes. Interaction with metal ions has some important consequences for the solubility, pharmacokinetics and bioavailability of quinolones, and is also involved in the mechanism of action of these bactericidal agents. Many metal complexes with equal or enhanced antimicrobial activity compared to the parent quinolones were obtained. New strategies in the design of metal complexes of quinolones have led to compounds with anticancer activity. Analytical applications of complexation with metal ions were oriented toward two main directions: determination of quinolones based on complexation with metal ions or, reversely, determination of metal ions based on complexation with quinolones.

## 1. Introduction

**Figure 1:**
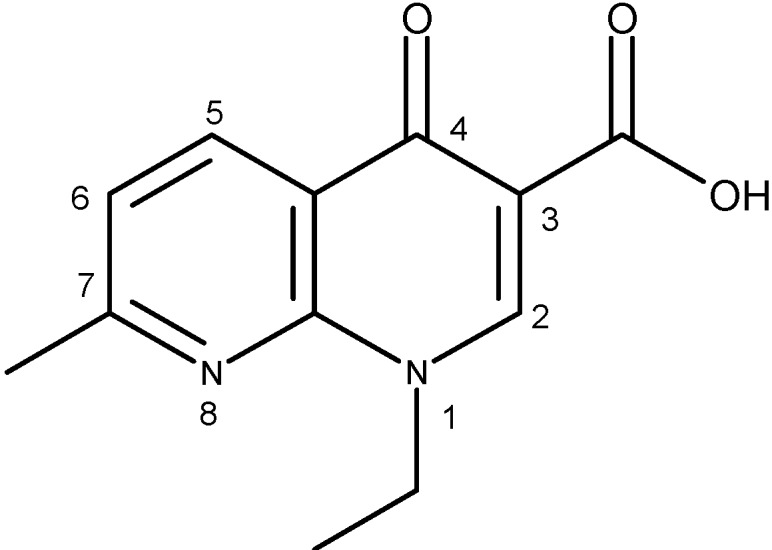
Nalidixic acid.

The generic term “quinolone antibiotics” refers to a group of synthetic antibiotics with bactericidal effects, good oral absorption and excellent bioavailability [1,2]. Nalidixic acid (1-ethyl-1,4-dihydro-7-methyl-4-oxo-1,8-naphthyridine-3-carboxylic acid, Figure 1), the first compound of the series, was introduced in therapy in the 1960s [3].

**Figure 2:**
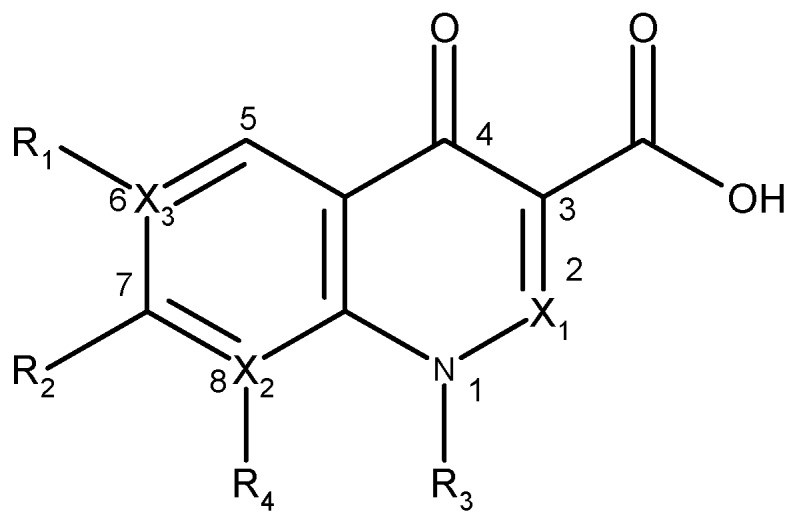
The general structure of 4-quinolones.

The clinical use of nalidixic acid was limited by its narrow spectrum of activity. Several modifications were made on the basis nucleus in order to enlarge the antibacterial spectrum and to improve the pharmacokinetics properties, two of these considered as being major: introduction of a piperazine moiety or another N-heterocycles in the position 7 and introduction of a fluoride atom at the position 6. Thus, the new 4-quinolones, fluoroquinolones, have been discovered starting in the 1980s. Taking into account the chemical structure of the basis nucleus (Figure 2), the quinolone are classified in four groups (Table 1) [4,5,6]. 

**Table 1:** Classes of quinolones based on chemical structure.

Quinolone group/base heterocycle	X_1_	X_2_	X_3_	R_1_	R_2_	R_3_	R_4_	Representatives	Generation
Naphthyridine (8-aza-4-quinolone)	CH	N	C	H	CH_3_	C_2_H_5_	-	Nalidixic acid	First
	CH	N	C	F	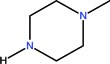	C_2_H_5_	-	Enoxacin	Second
	CH	N	C	F	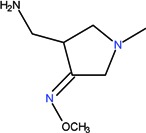	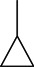	-	Gemifloxacin	Third
	CH	N	C	F	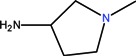	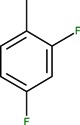	-	Tosufloxacin	Third
Pyridopyrimidine (6,8-diaza-4-quinolone)	CH	N	N	-	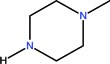	C_2_H_5_	-	Pipemidic acid	First
	CH	N	N	-	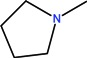	C_2_H_5_	-	Piromidic acid	First
Cinnoline (2-aza-4-quinolone)	N	C	C	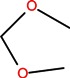		C_2_H_5_	H	Cinoxacin	First
Quinoline (4-oxo-1,4-dihydroquinoline, 4-quinolone)	CH	C	C	H	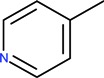	C_2_H_5_	H	Rosoxacin	First
	CH	C	C	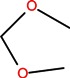		C_2_H_5_	H	Oxolinic acid	First
	CH	C	C	F	H	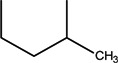		Flumequine	First
	CH	C	C	F	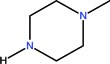	C_2_H_5_	H	Norfloxacin	Second
	CH	C	C	F	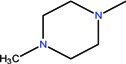	C_2_H_5_	H	Pefloxacin	Second
	CH	C	C	F	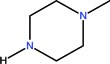	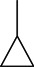	H	Ciprofloxacin	Second
	CH	C	C	F	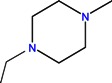	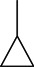	H	Enrofloxacin	Second
	CH	C	C	F	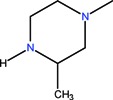	C_2_H_5_	F	Lomefloxacin	Second
	CH	C	C	F	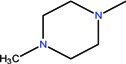	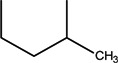		Ofloxacin	Second
	CH	C	C	F	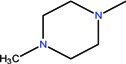	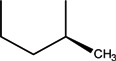		Levofloxacin	Third
	CH	C	C	F	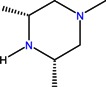	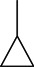	F	Sparfloxacin ^*^	Third
	CH	C	C	F	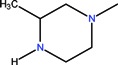	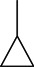	OCH_3_	Gatifloxacin	Third
	CH	C	C	F	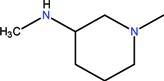	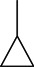	OCH_3_	Balofloxacin	Third
	CH	C	C	F	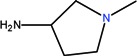	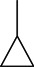	Cl	Clinafloxacin	Fourth
	CH	C	C	F	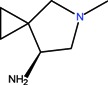	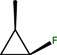	Cl	Sitafloxacin	Fourth
	CH	C	C	F	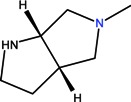	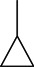	OCH_3_	Moxifloxacin	Fourth

* possesses a - NH_2_ group in position 5.

Based on their antibacterial spectrum and their pharmacokinetic properties, the quinolones are classified in four generations [7,8,9] (Table 2).

**Table 2:** Generations of quinolones based on their antibacterial spectrum and pharmacokinetic properties.

Quinolone generation	Characteristic features
**First**	Active against Gram negative bacteria.
	High protein binding.
	Short half life.
	Low serum and tissue concentrations.
	Uncomplicated urinary tract infection.
	Oral administration.
**Second **	**Class I** (enoxacin, norfloxacin, lomefloxacin)
	Enhanced activity against Gram negative bacteria.
	Protein binding (50%).
	Longer half life than the first generation.
	Moderate serum and tissue concentrations.
	Uncomplicated or complicated urinary tract infections.
	Oral administration.
	**Class II** (ofloxacin, ciprofloxacin)
	Enhanced activity against Gram negative bacteria.
	Atipical pathogens, Pseudomonas aeruginosa (ciprofloxacin).
	Protein binding (20%–50%).
	Moderate to long half life.
	Higher serum and tissue concentrations compared with class I.
	Complicated urinary infections, gastroenteritis, prostatitis, nosocomial infections.
	Oral and iv administration.
**Third**	Active against Gram negative and Gram positive bacteria.
	Similar pharmacokinetic profile as for second generation (class II).
	Similar indications and mode of administration. Consider for community aquired pneumonia in hospitalized patients.
**Fourth**	Extended activity against Gram positive and Gram negative bacteria.
	Active against anaerobes and atypical bacteria.
	Oral and i.v. administration.
	Consider for treatment of intraabdominal infections.

Quinolones are bactericidal agents that inhibit the replication and transcription of bacterial DNA, causing rapid cell death [10,11]. They inhibit two antibacterial key-enzymes, DNA-gyrase (topoisomerase II) and DNA topoisomerase IV. DNA-gyrase is composed of two subunits encoded as GyrA and GyrB, and its role is to introduce negative supercoils into DNA, thereby catalyzing the separation of daughter chromosomes. DNA topoisomerase IV is composed of four subunits, two ParC and two ParE subunits and it is responsible for decatenation of DNA thereby allowing segregation into two daughter cells [12,13]. Quinolones interact with the enzyme-DNA complex, forming a drug-enzyme-DNA complex that blocks progression and the replication process [14,15].

Older quinolones have greater activity against DNA-gyrase than against topoisomerase IV in Gram negative bacteria and greater activity against topoisomerase IV than against DNA-gyrase in Gram positive bacteria. Newer quinolones equally inhibit both enzymes [16,17,18].

## 2. Chemical Properties of Quinolones Related to Complexation Process

Most quinolone molecules are zwitterionic, based on the presence of a carboxylic acid function at the 3-position and a basic piperazinyl ring (or another N-heterocycle) at the 7-position. Both functions are weak and give a good solubility for the quinolones in acidic or basic media.

**Figure 3:**
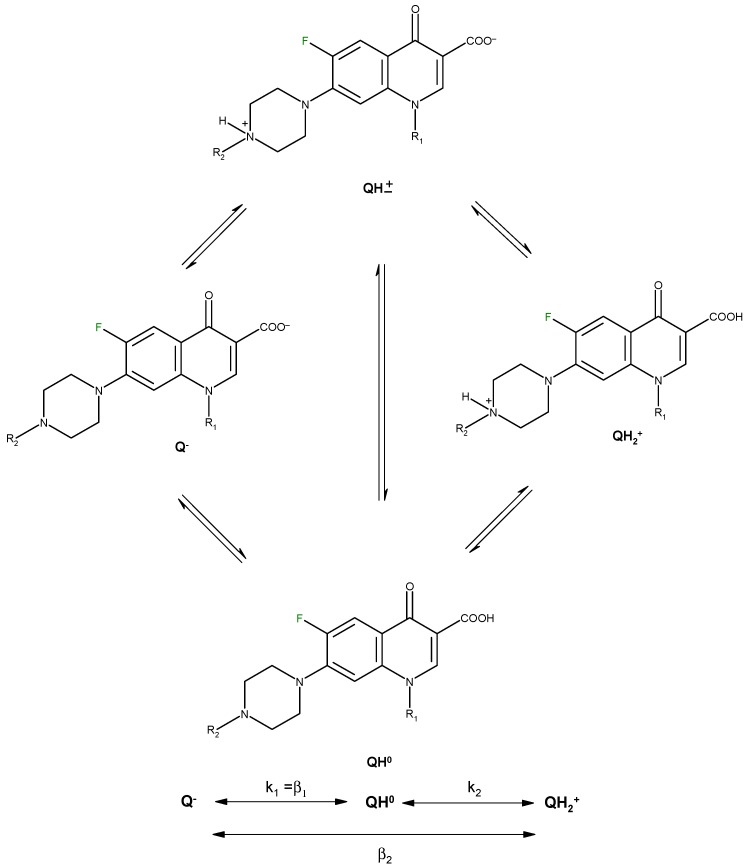
Protonation scheme of a fluoroquinolone molecule with piperazine ring at the 7-position (adapted from [20,21,22]).

Protonation equilibria of quinolones have been studied in aqueous solution using potentiometry, ^1^H- NMR spectrometry and UV spectrophotometry [19,20]. For a quinolone molecule with the general structure depicted in Figure 3, two proton-binding sites can be identified. In solution, such a molecule exists in four microscopic protonation forms, two of the microspecies being protonation isomers.

The microspeciation of drug molecules is used to depict the acid-base properties at the molecular level (macroconstants) and at the submolecular level (microconstants). The macroconstants quantify the overall basicity of the molecules. The values for pKa_1_, correlated with the acid function of carboxyl group, fall in the range 5.33–6.53, while the values for pKa_2_, correlated with the basic function of the piperazinic group, fall in the range 7.57–9.33. Table 3 contains the protonation constant values for norfloxacin and ofloxacin, two representative quinolones.

**Table 3:** Protonation constant values for norfloxacin and ofloxacin.

Compound	log β_1_	log β_2_ = log Ka_2_	log β_1_-log β_2_ = log Ka_1_	Isoelectric point	Reference
Norfloxacin	14.68	8.38	6.30	7.34	[19]
	14.73	8.51	6.22	7.37	[23]
Ofloxacin	14.27	8.22	6.05	7.14	[19]
	13.94	8.25	5.69	6.97	[23]

The microconstants describe the proton binding affinity of the individual functional groups and are used in calculating the concentrations of different protonation isomers depending on the pH. The quinolones exist mainly in the zwitterionic form between pH 3 and 11. The positively charged form QH^2+^ is present in 99.9% at pH 1. At pH 7.4 all microspecies are present in commensurable concentrations. 

Quinolone microspeciation has been correlated with bioavailability of quinolone molecules, serum protein binding and antibacterial activity [20]. The microspeciation is also important in the synthesis of metal complexes, the quinolone molecules acting as ligand in the deprotonated form (Q^−^) in basic conditions, and in the zwitterionic form (QH^±^) in neutral, slightly acidic or slightly basic medium. In strongly acidic medium, quinolones form ionic complexes in their cation form (QH_2_^+^). 

**Figure 4:**
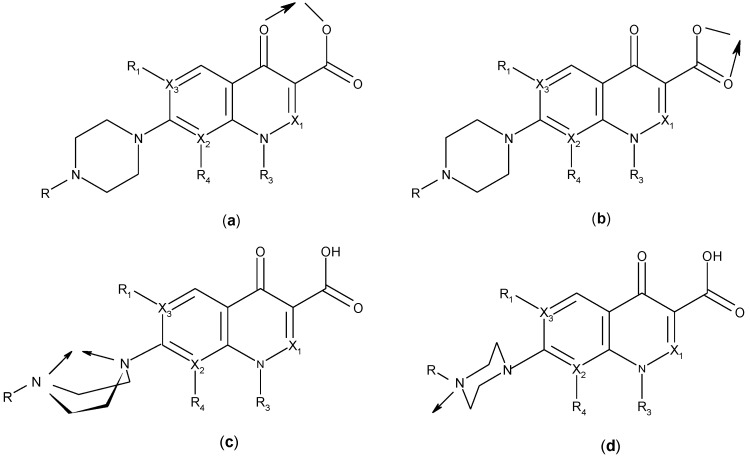
Main coordination modes of quinolones.

Quinolones form metal complexes due to their capacity to bind metal ions. In their metal complexes, the quinolones can act as bidentate ligand, as unidentate ligand and as bridging ligand. Frequently, the quinolones are coordinated in a bidentate manner, through one of the oxygen atoms of deprotonated carboxylic group and the ring carbonyl oxygen atom [Figure 4(a)]. Rarely, quinolones can act as bidentate ligand coordinated via two carboxyl oxygen atoms [Figure 4(b)] or through both piperazinic nitrogen atoms [Figure 4(c)]. Quinolones can also form complexes as unidentate ligand coordinated to the metal ion through by terminal piperazinyl nitrogen [Figure 4(d)]. In the polymeric complexes in solid state, multiple modes of coordination are simultaneously possible. In strongly acidic conditions quinolones are protonated and appear as cations in the ionic complexes.

## 3. Metal Complexes of Quinolones

### 3.1. Metal-Quinolone Chelates

**Figure 5:**
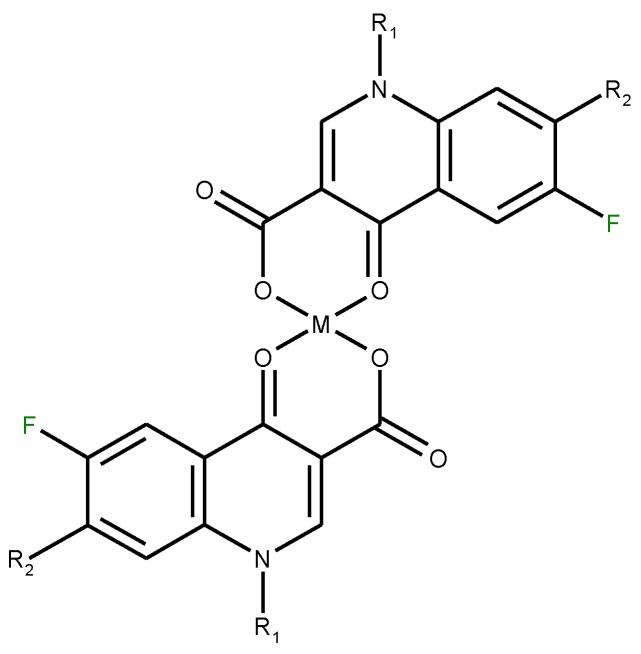
The general structure of 1:2 (metal:ligand) quinolone chelates with divalent cations.

The quinolone molecules possess two main sites of metal chelate formation [Figure 4(a,c)]. The first of these, represented by the carbonyl and carboxyl groups in neighboring positions, is the most common coordination mode in the quinolone chelates. Quinolones can bind divalent cations (Mg^2+^, Ca^2+^, Cu^2+^, Zn^2+^, Fe^2+^, Co^2+^ *etc*.), forming chelates with 1:1 or 1:2 (metal:ligand) stoichiometry or trivalent cations (A1^3+^, Fe^3+^), forming chelates with 1:1, 1:2 or 1:3 (metal:ligand stoichiometry). A higher stoichiometry (1:4) is found in complexes with Bi^3+^. In Figure 5 is depicted the general structure of the chelates of quinolones with divalent cations with the 1:2 (metal:ligand) molar ratio. In a study of the Cu(II)-ciprofloxacin system it was observed that the number of coordinated ligands depends on the pH. Thus, in the more acidic region, a 1:1 complex is favoured, whereas a 1:2 complex is the main species at higher pH values [24]. 

It was found that quinolones have a similar affinity for the metal ions, forming chelates more stable with hard Lewis acids like the trivalent cations (Al^3+^, Fe^3+^). Chelates less stable are formed with the cations of group 2A (Mg^2+^, Ca^2+^, Ba^2+^). For instance, the formation constant values for ciprofloxacin chelates decrease in order: Al^3+^ > Fe^3+^ > Cu^2+^ > Zn^2+^ > Mn^2+^ > Mg^2+^ [25]. For norfloxacin chelates, the variation is quite similar: Fe^3+^ > Al^3+^ > Cu^2+^ > Fe^2+^ > Zn^2+^ > Mg^2+^ > Ca^2+^ [26].

The stability of chelates is greater in solvents with lower dielectric constant [26] and is pH dependent; the affinity of lomefloxacin for the Ca^2+^ and Mg^2+^ ions decreases in the order: anion>zwitterion>>cation [27].

Table 4, Table 5 and Table 6 present a selection of the chelates obtained in solid state with quinolone acting as bidentate ligand through the pyridone oxygen and one carboxylate oxygen, and the type of experiments carried out for investigating their biological activity. The tables include those chelates in which the quinolones are the only bidentate ligands; complexes with other bidentate co-ligands (e.g., 2, 2'-bipyridine, 1,10-phenantroline), and their biological activity are not discussed here. 

**Table 4:** Selected chelates of quinolones from first generation.

Ligand	Metalion	Molar ratio M:L	General formulae of the complexes	Complex tested/investigated for	Reference
Pipemidic acid	VO^2+^	1:2	[VO(PPA)_2_(H_2_O)]	DNA binding antimicrobial activity	[28]
	Mn^2+^	1:2	[Mn(PPA)_2_(H_2_O)_2_]		
	Fe^3+^	1:3	[Fe(PPA)_3_]		
	Co^2+^	1:2	[Co(PPA)_2_(H_2_O)_2_]		
	Ni^2+^	1:2	[Ni(PPA)_2_(H_2_O)_2_]		
	Zn^2+^	1:2	[Zn(PPA)_2_(H_2_O)_2_]		
	MoO_2_^2+^	1:2	[MoO_2_(PPA)_2_]		
	Cd^2+^	1:2	[Cd(PPA)_2_(H_2_O)_2_]		
	UO_2_^2+^	1:2	[UO_2_(PPA)_2_]		
	Cu^2+^	1:2	[Cu(PPA)_2_(H_2_O)]	DNA binding antimicrobial activity	[29]
	Fe^3+^	1:1	[Fe (PPA)(HO)_2_(H_2_O)]_2_	-	[30]
Cinoxacin	Cu^2+^	1:2	[Cu(Cx)_2_(H_2_O)]·3H_2_O	*-*	[31]
	Ni^2+^		[Ni(Cx)_2_(DMSO)_2_]·4H_2_O		
	Cu^2+^	1:2	[Cu(Cx)_2_]·2H_2_O	antimicrobial activity	[32]
	Co^2+^	1:3	[Co(Cx)_3_]Na·10H_2_O	antimicrobial activity	[33]
	Cu^2+^	1:2	[Cu(Cx)_2_]·2H_2_O Cu(Cx)(HCx)Cl·2H_2_O		
	Zn^2+^	1:2	[Zn(Cx)_2_]·4H_2_O		
	Cd^2+^	1:1	Cd(Cx)Cl·H_2_O		
	Cd^2+^	1:3	Na_2_[(Cd(Cx)_3_)(Cd(Cx)_3_(H_2_O))] 12H_2_O	-	[34]
Oxolinic acid	Cu^2+^	1:2	[Cu(oxo)_2_(H_2_O)]	DNA binding antimicrobial activity	[35]
	Ni^2+^	1:2	[Ni(oxo)_2_(H_2_O)_2_]	DNA binding	[36]
	Zn^2+^	1:2	[Zn(oxo)_2_(H_2_O)_2_]	DNA binding	[37]
	VO^2+^	1:2	[VO(oxo)_2_(H_2_O)]	DNA binding	[38]
	Mn^2+^	1:2	[Mn(oxo)_2_(H_2_O)_2_]		
	Fe^3+^	1:3	[Fe(oxo)_3_]		
	Co^2+^	1:2	[Co(oxo)_2_(H_2_O)_2_]		
	Ni^2+^	1:2	[Ni(oxo)_2_(H_2_O)_2_]		
	Zn^2+^	1:2	[Zn(oxo)_2_(H_2_O)_2_]		
	Cd^2+^	1:2	[Cd(oxo)_2_(H_2_O)_2_]		
	MoO_2_^2+^	1:2	[MoO_2_(oxo)_2_]	DNA binding antimicrobial activity	[39]
	UO_2_^2+^	1:2	[UO_2_(oxo)_2_]		
Flumequine	Cu^2+^	1:2	[Cu(flmq)_2_(OH_2_)_2_]	-	[40]
	Zn^2+^		[Zn(flmq)_2_(OH_2_)_2_]·H_2_O		
	Cu^2+^	1:2	[Cu(flmq)_2_(H_2_O)]	DNA binding albumin binding	[41]
	Ni^2+^	1:2	[Ni(flmq)_2_(H_2_O)_2_]	DNA binding albumin binding	[42]
	Zn^2+^	1:2	[Zn(flmq)_2_(H_2_O)_2_]	DNA binding albumin binding	[43]

**Table 5:** Selected chelates of quinolones from second generation.

Ligand	Metal ion	Molar ratio M:L	General formulae of the complexes	Complex tested/ investigated for	Reference
Enoxacin	Co^2+^	1:2	[Co(HEx)_2_(ClO_4_)_2_]·3H_2_O [Co(HEx)_2_(NO_3_)_2_]·2H_2_O	antimicrobial activity DNA oxidative cleavage	[44]
	Cu^2+^ Ni^2+^	1:2	[M(Ex)_2_(H_2_O)_2_]·3H_2_O (M *=* Cu^II^, Ni^II^ or Mn^II^)	antimicrobial activity	[45]
	Mn^2+^ Fe^3+^		[Fe(Ex)(H_2_O)_2_]Cl·4H_2_O	antiinflammatory activity	
	Ni^2+^	1:2	Ni(Ex)_2_·2.5H_2_O	DNA binding	[46]
Norfloxacin	Mg^2+^	1:2	[M(Nf)_2_](ClO_4_)_2_·H_2_O	-	[47]
	Ca^2+^		M: Mg^2+^, Ca^2+^ (*n* = 4),		
	Ba^2+^		M: Ba^2+^ (*n* = 5)		
	Al^3+^	1:3	[(Nf·HCl)_3_Al]	solubility behavior	[48]
	Bi^3+^	1:4	[Bi (C_16_H_18_FN_3_O_3_)_4_(H_2_O)_2_]	antimicrobial activity solubility behavior	[49]
	Bi^3+^	1:3	[Bi(C_16_H_17_FN_3_O_3_)_3_(H_2_O)_2_]	antimicrobial activity, including Helicobacter pylori	[50]
	Mn^2+^	1:2	[M(Nf)_2_]X_2_·8H_2_O	-	[51]
	Co^2+^		(X = CH_3_COO^-^or SO_4_^2-^).		
	Fe^3+^	1:3	[Fe(Nf)_3_]Cl_3_·12H_2_O	-	
	Co^2+^	1:2	[Co(NfH-O,O’)_2_(H_2_O)_2_](NO_3_)_2_	-	[52]
	Mn^2+^ Co^2+^	1:1 1:1	[MnCl_2_(Nf)(H_2_O)_2_] [CoCl_2_(Nf)(H_2_O)_2_]	biological evaluation against Trypanosoma cruzi	[53]
	Ni^2+^	1:2	[Ni(Nf)_2_]·6H_2_O	DNA binding	[46]
	Cu^2+^	1:2	Cu(HNf)_2_·5H_2_O	-	[54]
			[Cu(HNf)_2_]Cl_2_·2H_2_O	-	
			Cu(HNf)_2_(NO_3_)_2_·H_2_O	-	
		1:2	[Cu(NfH)_2_]Cl_2_·6H_2_O	DNA binding albumin binding	[55]
	Zn^2+^	1:2	[Zn(Nf)_2_]·5H_2_O	-	[56]
	Zn^2+^ Cd^2+^ Hg^2+^	1:2	[M(Nf)_2_]X_2_·nH_2_O [M = Zn(II), (X = Cl^−^, CH_3_COO^−^, Br^−^ and I^−^), Cd(II), (X = Cl^−^, NO_3_^−^ and SO_4_^2−^) and Hg(II) (X = Cl^−^, NO_3_^−^ and CH_3_COO^−^)]	antimicrobial activity	[57]
	ZrO^2+^ UO_2_^2+^	1:2 1:3	[ZrO(Nf)_2_Cl]Cl·15H_2_O [UO_2_(Nf)_3_](NO_3_)_2_·4H_2_O	antimicrobial activity	[58]
	W^0^		[W(H_2_O)(CO)_3_(H-Nf)]· (H-Nf)·H_2_O	antimicrobial activity	[59]
	Ru^3+^	1:2	[Ru(Nf)_2_Cl_2_]·4H_2_O	-	[60]
	Pt^2+^	1:2	[Pt(Nf)_2_]	DNA binding DNA cleavage ability antimicrobial activity	[61]
	Au^3+^	1:1	[AuCl_2_(Nf)]Cl	DNA binding albumin binding cytotoxic activitycell cycle	[62]
	Y^3+^ Pd^2+^	1:2 1:2	[Y(Nf)_2_(H_2_O)_2_]Cl_3_·10H_2_O [Pd(Nf)_2_]Cl_2_·3H_2_O	antimicrobial activity	[63]
	La^3+^ Ce^3+^	1:3 1:3	[La(Nf)_3_]·3H_2_O [Ce(Nf)_3_]·3H_2_O	antimicrobial activity	[64]
	Ln= Nd(III) Sm(III) Ho(III)	1:4	[N(CH_3_)_4_][Ln(Nf)_4_]·6H_2_O	interaction with DNA and albumin	[65]
Pefloxacin	Bi^3+^	1:3	[Bi(C_17_H_19_FN_3_O_3_)_3_(H_2_O)_2_]	antimicrobial activity, including Helicobacter pylori	[50]
	Zn^2+^	1:2	[Zn (Pf)_2_(H_2_O)] ·2H_2_O	-	[66]
	Pt^2+^	1:2	[Pt(Pf)_2_]	DNA binding DNA cleavage ability antimicrobial activity	[61]
Ciprofloxacin	Mg^2+^	1:2	[Mg(Cf)_2_]·2.5H_2_O	DNA binding	[67]
	Mg^2+^	1:2	[Mg(Cf)_2_(H_2_O)_2_]·2H_2_O	antimicrobial activity	[68]
	Mg^2+^	1:2 1:3	[Mg(H_2_O)_2_(CfH)_2_](NO_3_)_2_·2H_2_O [Mg(CfH)_3_](SO_4_)·5H_2_O	-	[69]
	Mg^2+^ Ca^2+^ Ba^2+^	1:2	[M(Cf)_2_](ClO_4_)_2_·H_2_O M: Mg^2+^(*n* = 6) M: Ca^2+^ (*n* = 4) M: Ba^2+^(*n* = 2)	-	[47] [70]
	Mg^2+^ Zn^2+^ Co^2+^	1:2	[Mg(Cf)_2_(H_2_O)_2_]·2H_2_O [Zn(Cf)_2_]·3H_2_O [Co(Cf)_2_]·3H_2_O	*-*	[22]
	Al^3+^	1:3	[(Cf·HCl)_3_Al]		[48]
	Bi^3+^	1:3	[Bi(C_17_H_17_FN_3_O_3_)_3_(H_2_O)_2_]	antimicrobial activity, including Helicobacter pylori	[50]
	VO^2+^	1:2	[VO(Cf)_2_(H_2_O)]	-	[71]
	Mn^2+^ Co^2+^ Ni^2+^ Cu^2+^ Zn^2+^ Cd^2+^	1:1	[Mn(Cf)(OAc)(H_2_O)_2_]·3H_2_O [Co(Cf)(OAc)(H_2_O)_2_]·3H_2_O [Ni(Cf)(OAc)]·6H_2_O [Cu(Cf)(OAc)(H_2_O)_2_]·3H_2_O [Zn(Cf)(OAc)]·6H_2_O [Cd(Cf)(OAc)(H_2_O)_2_]·3H_2_O	antimicrobial activity	[72]
	Mn^2+^ Fe^3+^, Co^2+^ Ni^2+^ MoO_2_^2+^	1:2 for M^2+^ 1:3 for Fe^3+^	[Mn(Cf)_2_(H_2_O)_2_] [Fe(Cf)_3_] [Co(Cf)_2_(H_2_O)_2_] [Ni(Cf)_2_(H_2_O)_2_] [MoO_2_(Cf)_2_]	DNA binding	[73]
	Co^2+^ Zn^2+^ Cd^2+^ Ni^2+^ Cu^2+^	1:2	[Co(Cf)_2_(H_2_O)]·9H_2_O [Zn(Cf)_2_(H_2_O)_2_]·8H_2_O [Cd(HCf)_2_(Cl)_2_ ]·4H_2_O M(Cf)_2_·xH_2_O [M = Ni, Cu, Cd]	antimicrobial activity	[34]
	Co^2+^	1:2	[Co(Cf)_2_]·3H_2_O	*-*	[22]
	Cu^2+^	1:2	[Cu(HCf)_2_](NO_3_)_2_]·6H_2_O	-	[74]
	Cu^2+^	1:2	[Cu(Cf)_2_]Cl_2_·11H_2_O	-	[75]
	Cu^2+^	1:2	[Cu(Cf)_2_]Cl_2_·6H_2_O	-	[76]
	Cu^2+^	1:2	[Cu(HCf)_2_(ClO_4_)_2_]·6H_2_O [Cu(HCf)_2_(NO_3_)_2_]·6H_2_	antimicrobial activity	[44]
		1:1	[Cu(HCf)(C_2_O_4_)]·2H_2_O	DNA oxidative cleavage	[44]
	Cu^2+^/ Cu^+^	3:2	[Cu^II^(Cf)_2_(Cu^I^Cl_2_)_2_]	antimicrobial activity Gyrase inhibition DNA cleavage	[77]
	Ru^3+^	1:2	[Ru(Cf)_2_Cl_2_]Cl·3H_2_O	-	[60]
		1:3	[Ru(Cf)_3_]·4H_2_O	DNA interaction	[78]
	Pd^2+^	1:1	[PdCl_2_(L)]	antitubercular activity	[79]
	Eu^3+^	1:2	[Eu(CfH)(Cf)(H_2_O)_4_]Cl_2_·4.55H_2_O	-	[80]
Lomefloxacin	Bi^3+^	1:3	[Bi(C_17_H_18_F_2_N_3_O_3_)_3_(H_2_O)_2_]	antimicrobial activity, including H. pylori	[50]
	Y^3+^	1:2	[Y(LFX)_2_Cl_2_]Cl·12H_2_O	antimicrobial activity	[81]
	ZrO^2+^	1:2	[ZrO(LFX)_2_Cl]Cl·15H_2_O		
	UO_2_^2+^	1:3	[UO_2_(LFX)_3_](NO_3_)_2_·4H_2_O		
	Cr^3+^	1:1	[Cr(LFX)(H_2_O)_4_]Cl_3_	antimicrobial, antifungal, and anticancer activity	[82]
	Mn^2+^	1:1	[Mn(LFX)(H_2_O)_4_]Cl_2_		
	Fe^3+^	1:1	[Fe(LFX)(H_2_O)_4_]Cl_3_·H_2_O		
	Co^2+^	1:1	[Co(LFX)(H_2_O)_4_]Cl_2_		
	Ni^2+^	1:1	[Ni(LFX)(H_2_O)_4_]Cl_2_·H_2_O		
	Cu^2+^	1:1	[Cu(LFX)(H_2_O)_4_]Cl_2_·2H_2_O		
	Zn^2+^	1:1	[Zn(LFX)(H_2_O)_4_]Cl_2_		
	Th(IV)	1:1	[Th(LFX)(H_2_O)_4_]Cl_4_		
	UO_2_^2+^	1:1	[UO_2_(LFX)(H_2_O)_2_](NO_3_)_2_		
Ofloxacin	Mg^2+^	1:2	[Mg(R-oflo)(S-oflo)(H_2_O)_2_]·2H_2_O	antimicrobial activity	[83]
	Ca^2+^	1:1	Ca(oflo)Cl·2H_2_O	-	[84]
	Mg^2+^		Mg(oflo)Cl·2H_2_O		
	Ba^2+^		Ba(oflo)Cl·2H_2_O		
	Ni^2+^		Ni(oflo)Cl·2H_2_O		
	Co^2+^		Co(oflo)Cl·2H_2_O		
	Zn^2+^		Zn(oflo)Cl·H_2_O		
	Cu^2+^	1:2	[Cu^II^(ofloH)_2_][(Cu^I^Cl_2_)_2_]	DNA binding albumin binding	[55]
	Co^2+^ Zn^2+^	1:2	[M(oflo)_2_]·4H_2_O	-	[85]
	Cu^2+^	1:1	M(oflo)Cl·2.5H_2_O	-	[86]
	Ni^2+^		M(oflo)(SO_4_)_0.5_·2.5H_2_O		
			M(oflo) (NO_3_)·2.5H_2_O		
		1:2	[Cu(oflo)_2_·H_2_O]·2H_2_O		
			Ni(oflo)_2_·3H_2_O		
	Pd^2+^	1:1	[PdCl_2_(L)]	antitubercular activity	[79]
	Pt^2+^	1:2	[Pt(oflo)_2_]	DNA binding antimicrobial activity	[61]
	Bi^3+^	1:3	[Bi(C_17_H_17_FN_3_O_3_)_3_(H_2_O)_2_]	antimicrobial activity, including Helicobacter pylori	[50]
	Pr^3+^ Nd^3+^	1:1	[PrL(NO_3_)_2_(CH_3_OH)](NO_3_) [NdL(NO_3_)_2_(CH_3_OH)](NO_3_)	DNA binding DNA cleavage activity antioxidation properties	[87]
Enrofloxacin	VO^2+^	1:2	[VO(erx)_2_(H_2_O)]	antimicrobial activity DNA binding	[88]
	MO_2_^2+^	1:2	[MoO_2_(erx)_2_]	antimicrobial activity DNA binding	[89]
	Mn^2+^ Fe^3+^ Co^2+^ Ni^2+^ Zn^2+^ Cd^2+^ UO_2_^2+^	1:2 for M^2+^, 1:3 for Fe^3+^	[Mn(erx)_2_(H_2_O)_2_] [Fe(erx)_3_] [Co(erx)_2_(H_2_O)_2_] [Ni(erx)_2_(H_2_O)_2_] [Zn(erx)_2_(H_2_O)_2_] [Cd(erx)_2_(H_2_O)_2_] [UO_2_(erx)_2_]	antimicrobial activity DNA binding	[90]
	Ni^2+^	1:2	[Ni(erx)_2_(H_2_O)_2_]	DNA binding albumin binding	[91]
	Cu^2+^	1:2	[Cu(erx)_2_]Cl	antimicrobial activity	[92]
	Cu^2+^	1:2	[Cu(erx)_2_(H_2_O)]	DNA binding antimicrobial activity	[93]
	Cu^2+^	1:2	[Cu(erx)_2_(H_2_O)_2_]	-	[94]
	Ru^3+^	1:2	[Ru(erx)_2_Cl_2_]Cl·5H_2_O	-	[60]

**Table 6:** Selected chelates of quinolones from third and fourth generation.

Ligand	Metal ion	Molar ratio M:L	General formulae of the complexes	Complex tested/investigated for	Reference
Sparfloxacin	Bi^3+^	1:3	[Bi(C_19_H_21_F_2_N_4_O_3_)_3_(H_2_O)_2_]	antimicrobial activity, including Helicobacter pylori	[50]
	Fe^3+^, VO^2+^ Mn^2+^ Ni^2+^ UO_2_^2+^	1:3 1:2 for M^2+^	[Fe(sf)_3_] [VO(sf)_2_(H_2_O)] [Mn(sf)_2_(H_2_O)_2_] [Ni(sf)_2_(H_2_O)_2_] [UO_2_(sf)_2_]	DNA binding Serum albumin binding	[95]
	Co^2+^	1:2	[Co(sf)_2_(H_2_O)_2_]	antimicrobial activity DNA binding	[96]
	Cu^2+^	1:2	[Cu(sf)_2_]	antimicrobial activity DNA binding	[97]
	Mn^2+^ Co^2+^	1:1 1:1	[MnCl_2_(sf)(H_2_O)_2_] [CoCl_2_(sf)(H_2_O)_2_]	biological evaluation against Trypanosoma cruzi	[53]
	MO_2_^2+^	1:2	[MoO_2_(sf)_2_]	antimicrobial activity DNA binding	[89]
	Pd^2+^	1:1	[PdCl_2_(L)]	antitubercular activity	[79]
	Pt^2+^	1:2	[Pt(sf)_2_]	DNA bindingDNA cleavage abilityantimicrobial activity	[61]
	Au^3+^	1:1	[AuCl_2_(sf)]Cl	DNA bindingalbumin bindingcytotoxic activitycell cycle	[62]
Levofloxacin	Mg^2+^	1:2	[Mg(S-oflo)_2_(H_2_O)_2_]·2H_2_O	antimicrobial activity	[83]
	Mn^2+^ Co^2+^ Ni^2+^ Cu^2+^ Zn^2+^	1:2	[M(levo)_2_(H_2_O)_2_]·nH_2_O (*n* = 2, excepting for Cu^2+^, *n* = 3)	antimicrobial activity immunomodulatory activity cytotoxicity	[98]
	Zn^2+^	1:2	[Zn(levo)_2_(H_2_O)_2_]	DNA binding albumin binding	[99]
	Pd^2+^	1:1	[PdCl_2_(L)]	antitubercular activity	[79]
	Pt^2+^	1:2	[Pt(levo)_2_]	DNA binding DNA cleavage ability antimicrobial activity	[61]
	Au^3+^	1:1	[AuCl_2_(levo)]Cl	DNA binding albumin binding cytotoxic activity cell cycle	[62]
Gatifloxacin	Mg^2+^ Ca^2+^ Cr^3+^ Mn^2+^ Fe^3+^ Co^2+^ Ni^2+^ Cu^2+^ Zn^2+^ Cd^2+^	1:2	[Mg(gat)_2_(H_2_O)_2_]Cl_2_·2H_2_O [Ca(gat)_2_(H_2_O)_2_]Cl_2_·2H_2_O [Cr(gat)_2_ Cl(H_2_O)_2_]Cl·2H_2_O [Mn (gat)_2_(H_2_O)_2_]·6H_2_O [Fe(gat)_2_Cl(H_2_O)_2_]Cl·2H_2_O [Co (gat)_2_(H_2_O)_2_]·4H_2_O [Ni (gat)_2_(H_2_O)_2_] Cl_2_·2H_2_O [Cu (gat)_2_(H_2_O)_2_]·H_2_O [Zn (gat)_2_(H_2_O)_2_]·2H_2_O [Cd (gat)_2_(H_2_O)_2_] Cl_2_·4H_2_O	antimicrobial activity antifungal activity antiiinflamatory	[100]
	Zn^2+^ Ni^2+^ Co^2+^	1:2	[M(gat)_2_(H_2_O)_2_]·4H_2_O	antimicrobial activity	[101]
	Bi^3+^	1:3	[Bi(C_19_H_21_FN_3_O_4_)_3_(H_2_O)_2_]	antimicrobial activity, including Helicobacter pylori	[50]
	Pd^2+^	1:1	[PdCl_2_(L)]	-	[79]
	Pt^2+^	1:2	[Pt(gat)_2_]	DNA binding DNA cleavage ability antimicrobial activity	[61]
	Rh^3+^	1:1	[X]^+^ *fac*-[RhCl_3_(L)(gat)]^-^ where L = H_2_O, Dimethylsulfoxide (DMSO), Tetramethylenesulfoxide (TMSO); gat = Gatifloxacin and X = Na or [H(DMSO)_2_].	antimicrobial activity	[102]
Moxifloxacin	Cu^2+^	1:1	[Cu(MOX)(H_2_O)_2_Cl]BF_4_	anti-proliferative and apoptosis-inducing activity	[103]
	Pd^2+^ Y^3+^ Ti(IV) Ce(IV)	1:2 1:2 1:2 1:2	[Pd(MOX)_2_(H_2_O)_2_]Cl_2_·6H_2_O [Y(MOX)_2_Cl_2_]Cl·12H_2_O [Ti(MOX)_2_](SO_4_)_2_·7H_2_O [Ce(MOX)_2_](SO_4_)_2_·2H_2_O	antimicrobial activity	[104]
	VO^2+^ Zr(IV) UO_2_^2+^	1:2 1:2 1:3	[VO(MOX)_2_H_2_O]SO_4_·11H_2_O [ZrO(MOX)_2_Cl]Cl·15H_2_O [UO_2_(MOX)_3_](NO_3_)_2_·3H_2_O	antimicrobial activity	[105]

The first review regarding the interactions of metal ions with quinolone was published ten years ago and discussed selected crystal structures of quinolone–metal compounds, different physico-chemical methods of characterization, as well as some results of bioactivity test [21]. The structural characteristics of a part of fluoroquinolone complexes and their biological activity were reviwed four years ago [106]. A recent comprehensive review [107] presented the structures and the biological activity of complexes of some quinolones with Cu(II), Ni(II), Co(II) and Zn(II) and analysed the influence of the second ligand on biological activity. 

**Figure 6:**
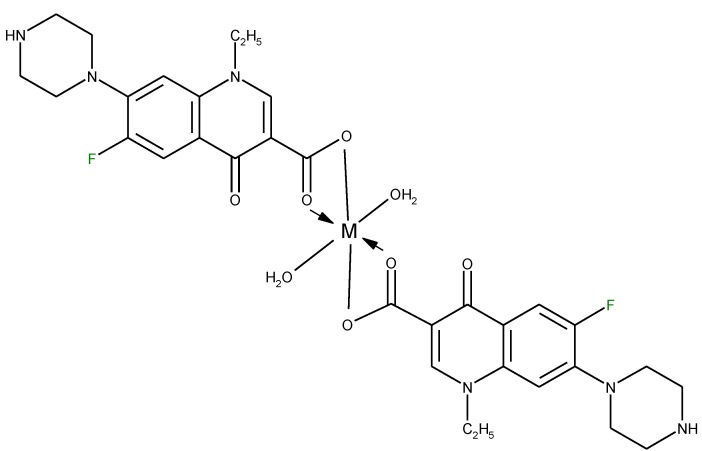
The proposed structure of complexes of Fe(III)-Nf and Co(II)-Nf (adapted from [108]).

**Figure 7:**
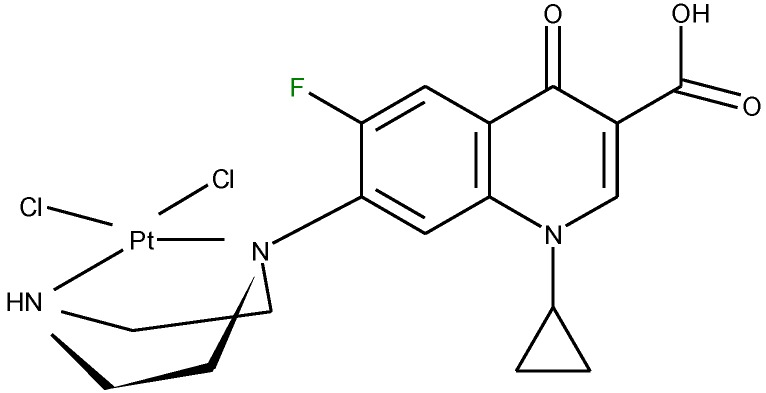
Proposed structure for [PdCl_2_(L)] (adapted from [104]).

In one report, norfloxacin acts as bidentate ligand through two carboxylate oxygen atoms (Figure 6) in complexes with Co(II) and Fe(III) ions [108]. A quite rare coordination mode of quinolones occurs in a bidentate fashion via the piperazine nitrogen atoms. This coordination was reported in complexes of general formula [PtCl_2_(L)] (Figure 7) formed by ciprofloxacin, levofloxacin, ofloxacin, sparfloxacin, and gatifloxacin with Pt(II) [109], and could be explained through the basicity both of N4 nitrogen from piperazine ring and of N1 nitrogen, the last one evidenced in recent studies [110]. 

### 3.2. Chelates Introduced into the Polyoxometalates (POMs) Surface

Quinolone molecules are excellent multidentate ligands able to construct metal–organic polymers with medical applications, due to the higher electronic cloud density of oxygen and nitrogen atom [111]. Such hybrid organic-inorganic materials have been obtained by introducing a quinolone chelate into the surface of a polyoxometalate anion. The polyoxometalates (POMs) are known as anti-tumor, antiviral, and antibacterial inorganic medical agents, and the modifying of their surface with such compounds with biological activity is aimed to ameliorate their properties.

Generally, these complexes were obtained by hydrothermal reaction of a quinolone with a metal salt and a polyoxometalate (in the acidic form or as ammonium salt) with adjusting the pH.

One of the simplest compound of this series is V_4_O_10_(μ_2_-O)_2_[VO(H-Cf)_2_)]_2_·13H_2_O, with a structure consisting in one {V_4_O_12_} unit and two corner-sharing octahedral {VO_6_}-ciprofloxacin units linked through two μ_2_-O bridges [112].

**Figure 8:**
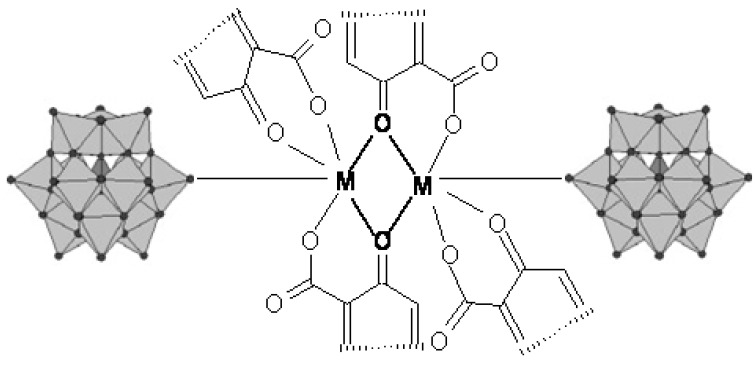
A binuclear metallic cluster of quinolone bound to POM clusters.

**Figure 9:**
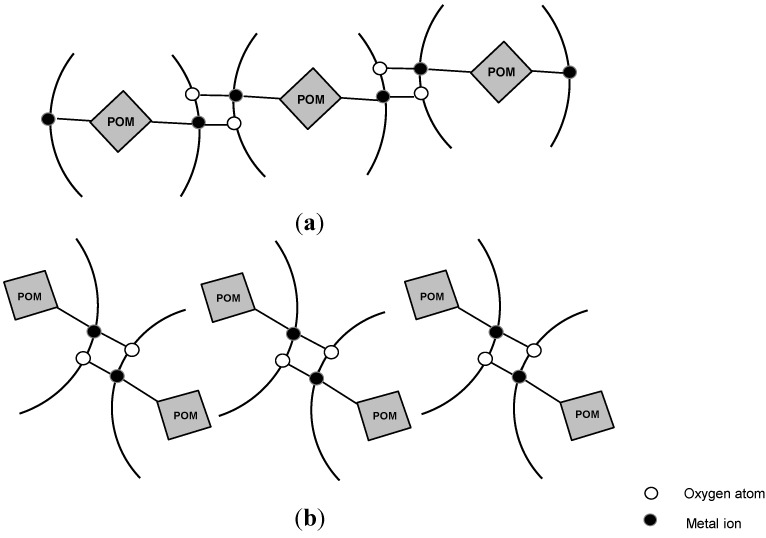
Schematic representation of the 1D chain structure, constructed by POMs and M-quin binuclear clusters with POM bound as (**a**) bidentate bridging ligand or (**b**) unidentate ligand.

Anions with α-Keggin structure (PW_12_O_40_^4-^, SiW_12_O_40_^4-^) were used as inorganic building blocks in compounds constructed from PW_12_ or SiW_12_ clusters and two M(Quin)_2_ chelates. The PW_12_ or SiW_12_ clusters and quinolone molecule as chelating bidentate organic ligands coordinate the metal ions together (Figure 8). The binuclear metal clusters are connected to the POM clusters, bound as unidentate or as bridging bi-dentate inorganic ligands, forming a 1D chain architecture, as shown in Figure 9. 

Starting to polyoxometalates (POMs) and the quinolone antibacterial drug pipemidic acid (HPPA), complexes as {[Co(PPA)_2_]H_2_[SiW_12_O_40_]}∙HPP∙3H_2_O [113], [Cu(PPA)_2_]_2_·[PW_12_O_40_]∙6H_2_O [114], {[Ni(PPA)_2_]H_4_[SiW_12_O_40_]}∙HPPA∙3H2O, and {[Zn(PPA)_2_]_2_H_4_[SiW_12_O_40_]}∙3H_2_O [115] were obtained. By introducing different quinolone antibacterial drugs into the octamolybdate POMs new compounds have been isolated, such as [Cu^II^(L^1^)_2_(H_2_O)_2_]H_2_[β-Mo_8_O_26_]∙4H_2_O (1), [Cu^II^_2_(L^2^)_4_][δ-Mo_8_O_26_]∙4H_2_O (2), [Cu^II^_2_(L^3^)_2_(H_2_O)_2_][β-Mo_8_O_26_] (3), [Cu^II^_2_(L^4^)_2_(H_2_O)_4_][β-Mo_8_O_26_]∙2H_2_O (4) (where L^1^ = enrofloxacin; L^2^ = pipemidic acid; L^3^ = norfloxacin; L^4^ = enoxacin) [111].

### 3.3. Metal Complexes with Quinolone Acting as Unidentate Ligand

**Figure 10:**
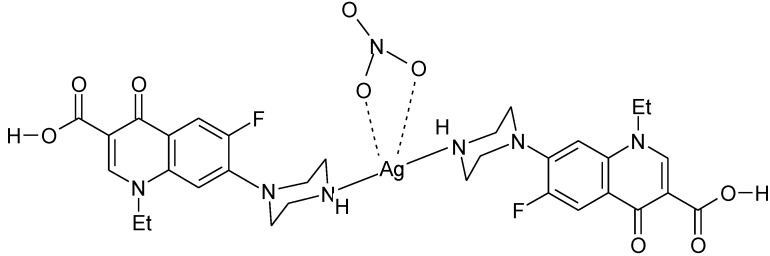
Proposed structure for the complex Ag(H-Nf)_2_(NO_3_) [116].

The quinolones bearing a piperazinyl ring in the 7-position could form complexes where the terminal piperazinyl nitrogen (N4) is involved in the coordination to the metal ion. This coordination mode was reported for complexes with transition metals Ag(I), Au(III), and Ru(III). The structure proposed for the complex Ag_2_(Nf)_2_(NO_3_)_2_ [116] is presented in Figure 10.

**Figure 11:**
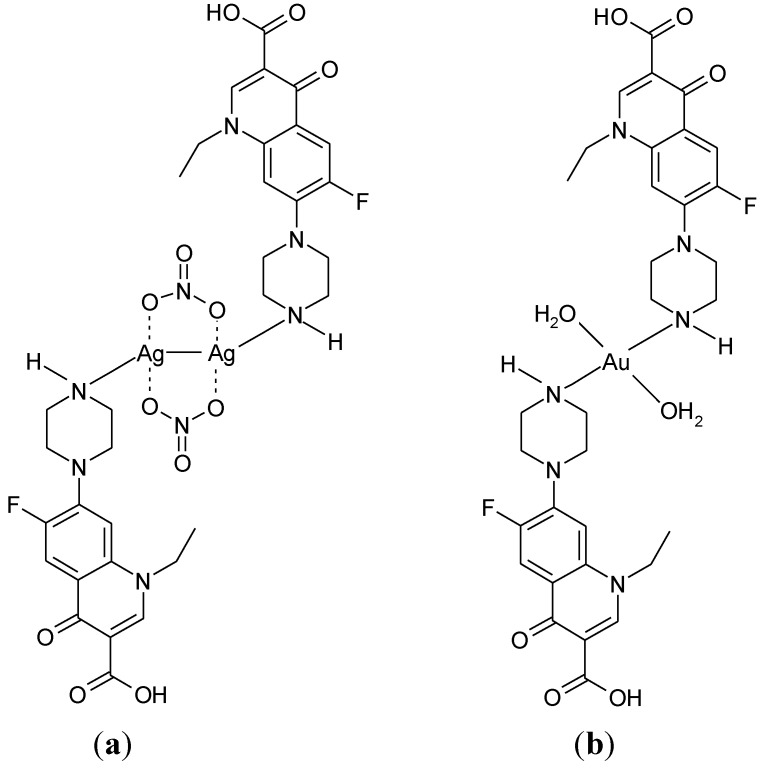
Proposed structures for (**a**) Ag_2_(Nf)_2_(NO_3_)_2_, and (**b**) [Au(Nf)_2_(H_2_O)_2_]Cl_3_ [117].

By the reaction of Ag(I) and Au(III) with norfloxacin, a dinuclear complex Ag_2_(Nf)_2_(NO_3_)_2_ [Figure 11(a)], and a mononuclear complex [Au(Nf)_2_(H_2_O)_2_]Cl_3_ [Figure 11(b)] were obtained [117].

In some complexes of Ru(III), formulated as Ru(L)_2_Cl_3_(DMSO)_m_∙xH_2_O (L: pipemidic acid, enoxacin, enrofloxacin, ciprofloxacin, norfloxacin, ofloxacin, levofloxacin), quinolones are bound as unidentate ligand coordinate through the N4 piperazinyl nitrogen [118,119]. 

### 3.4. Polymeric Complexes

**Figure 12:**
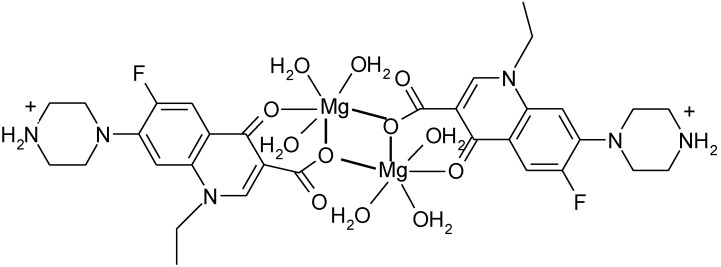
Structure of the dimeric complex [Mg_2_(H_2_O)_6_(HNf)_2_]Cl_4_·4H_2_O (adapted from [120]).

Dimeric complexes [Mg_2_(H_2_O)_6_(HNf)_2_]Cl_4_·4H_2_O and [Ca_2_(Cl)(HNf)_6_]Cl_3_·10H_2_O [120] are formed with norfloxacin as bidentate bridging ligand bound through the pyridone oxygen and one carboxylate oxygen atom (unidentate bridging) (Figure 12).

**Figure 13:**
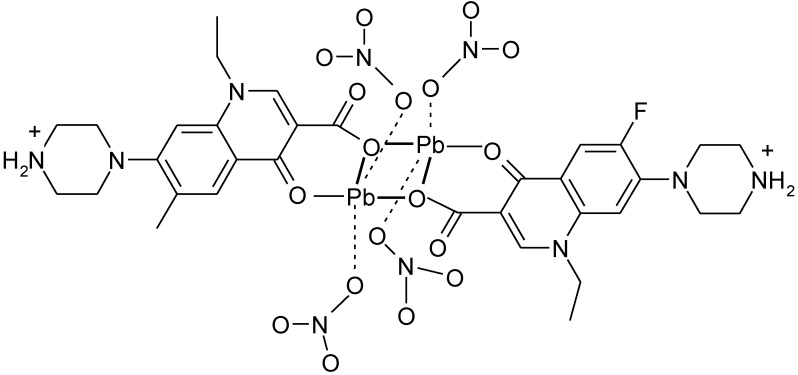
Structure of the dimeric complex [Pb(H-Nf)(ONO_2_)_2_]_2_ (adapted from [121]).

A similar coordination it was found in the complex [Pb(H-Nf)(ONO_2_)_2_]_2_ (Figure 13) [121].

X-ray determination of crystal structure of the dinuclear complexes [Cd_2_(Cx)_4_(H_2_O)_2_]·10H_2_O and [Cd_2_(Cx)_4_(DMSO)_2_]·2H_2_O revealed that the cadmium ion is heptacoordinated; the coordination environment consists in two cinoxacinate ions acting as tridentate chelate and bridging ligands, one as bidentate chelate ligand, and one water molecule [33].

**Figure 14:**
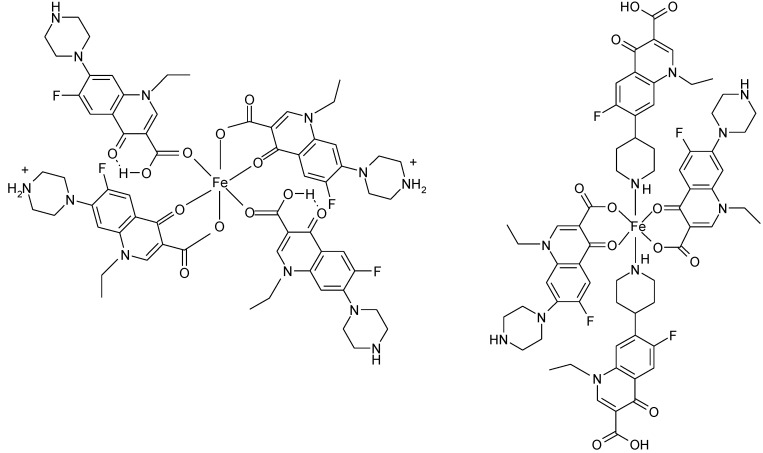
Coordination modes of norfloxacin in (**a**) Fe(H-Nf)_2_(SO_4_)·2H_2_O and (**b**) Fe(Nf)_2_·4H_2_O (adapted from [122]).

In polymeric complexes, different modes of coordination are simultaneously possible. In the case of two Fe(II) complexes, norfloxacin adopts different modes of coordination depending on the synthesis conditions. In the structure of Fe(H-Nf)_2_(SO_4_)·2H_2_O, Fe(II) is surrounded by two norfloxacinate anions bound as bidentate ligand coordinated through the pyridone oxygen and one carboxyl carboxylate oxygen and two norfloxacin molecules coordinated as unidentate ligand by two oxygen atoms from two different carboxylate [Figure 14(a)]. In the other complex, Fe(Nf)_2_·4H_2_O, two molecules are bound as bidentate ligand, and two as unidentate ligand coordinated through piperazine nitrogen [Figure 14(b)] [122].

**Figure 15:**
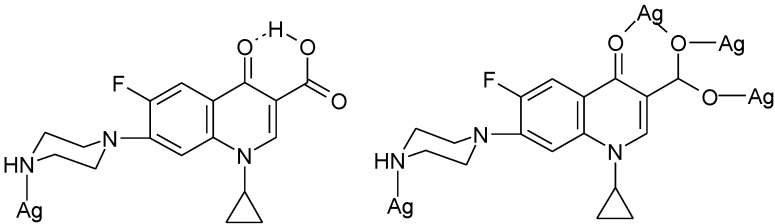
Coordination modes of ciprofloxacin and its anion in {[Ag_4_(H-Cf)_2_(Cf)_2_(NO_3_)_2_]·4H_2_O}_n_ [123].

In a 1D ladder-like silver(I) coordination polymer, {[Ag_4_(H-Cf)_2_(Cf)_2_(NO_3_)_2_]·4H_2_O}_n_ [123] the pseudo-tetranuclear building blocks are constructed via unidentate ciprofloxacin coordinated through the N4 piperazine atom and tetradentate deprotonated ciprofloxacin ligands (Figure 15).

### 3.5. Ionic Complexes

Based on the basic function of the N4 pyperazinyl atom, quinolones are protonated in acidic medium, forming ionic chlometalates, generally obtained by slow evaporation of an acidic solution of complex and metal salt. Most of these complexes were tested for their antimicrobial activity (see Subsection 4.3).

The chloroantimonates (III) obtained with nalidixium C_12_H_13_N_2_ (nalidixium cation) and ciprofloxacinium ions have the general formulae (C_12_H_13_N_2_O_3_)[SbCl_4_]·H_2_O [124], and (C_17_H_19_N_3_O_3_F) [SbCl_5_]·H_2_O (ciprofloxacinium cations (CfH_3_)^2+^) [125] respectively. Two types of chlorobismutates (III) were obtained with ciprofloxacin, (CfH_2_)(CfH)[BiCl_6_]·2H_2_O [126] and (CfH_2_)_2_[Bi_2_Cl_10_]·4H_2_O [127].

The tetrachlorocuprates (II) synthesized from norfloxacin, pefloxacin, and cinoxacin, were formulated as (NfH_2_)(NfH)[CuCl_4_]Cl·H_2_O [128], (C_17_H_22_FN_3_O_3_)^2+^[CuCl_4_]^2−^ [129], and (CxH_2_)[CuCl_4_]·H_2_O [129], respectively.

Other chloromethalates, such as enrofloxacinium tetrachloroferate (II), (erxH_2_)[FeCl_4_]Cl [130], ciprofloxacinium tetrachlorozincate (II) dihydrate, [C_17_H_19_N_3_O_3_F]_2_[ZnCl_4_]·2H_2_O [131], ciprofloxacinium tetrachloroaurate (III) monohydrate, (CfH_2_)[AuCl_4_]· H_2_O [132] and ciprofloxacinium hexachlororuthenate (III) trihydrate, (CfH_2_^+^)_3_[RuCl_6_]·3H_2_O [78] were also reported.

## 4. Consequences and Applications of Metal-Quinolone Complexation

### 4.1. Pharmaceutical Aspects

Some chelates of quinolones with trivalent cations have shown an improved solubility compared to that of the free ligand, and this behaviour could be advantageous for pharmaceutical formulation. The hydrochlorides of the aluminium (III) complexes of ciprofloxacin and norfloxacin were reported [48,133]. Both complexes are more soluble than the antibiotics themselves. The complexes can be used for developing more dose-efficient formulations, such as compressed tablet dosage forms [48,134]. The pharmacodynamic properties of ciprofloxacin are not drastically affected upon complexation with aluminium. The complex [(HCl·Cf)_3_Al] showed a longer post-antibiotic effect (PAE) compared to that the free ciprofloxacin [135]. 

The solubility studies of a bismuth (III) complex of norfloxacin, [Bi(C_16_H_18_FN_3_O_3_)_4_(H_2_O)_2_] (BNC) in different pH buffers indicated that the solubility of the BNC was higher than that of norfloxacin until pH 6.5. Above this pH value, a significant decrease in the solubility of BNC was observed, while the solubility of norfloxacin did not change significantly. The increased solubility can be an advantage for the antibacterial activity of the bismuth complex [49].

### 4.2. Biopharmaceutical and Pharmacokinetic Implications

Reducing the oral bioavailability of quinolones in the presence of multivalent cations is the main consequence of the metal ions-quinolones interaction, and it was reported for the first time in 1985 [136]. A reduction in ciprofloxacin biavailability in healthy human subjects was observed at co-administration with ferrous salts and a combination of multi-vitamin and mineral preparation. In correlation with UV-Vis spectra features, the formation of a 1:3 ferric ion-ciprofloxain complex was proposed as the cause of the reduction in ciprofloxacin biovailability [137]. A strong correlation between the reduction in oral bioavailability of norfloxacin in the presence of divalent and trivalent cations and the magnitude of formation constants measured *in vitro* was established (Ca^2+^ < Mg^2+^ < Zn^2+^ ~ Fe^2+^ < Al^3+^). A marked difference between the effect of Zn^2+^ and Fe^2+^ was observed *in vivo*, namely a greater reduction in norfloxacin absorption with co-administration of Fe^2+^. The oxidation of Fe^2+^ to Fe^3+^ in gastrointestinal tract was proposed as possible explanation [138]. 

Several mechanisms were proposed in order to explain the decreased biovailability of quinolone in the presence of metal ions. The first hypothesis was that the reduction of quinolone absorption is due to the formation of insoluble and unabsorbable chelates in the gastrointestinal tract [139,140,141]. On the contrary, in other studies it was observed that the solubility of lomefloxacin increases in the presence of Ca^2+^, Mg^2+^, Al^3+^ şi Fe^3+^ ions [142]. This means that the reduction of the gastric absorption of lomefloxacin at co-administration with these metal ions, are not caused by the precipitation, but by a decrease of the octanol-water partition cofficient. Only for Bi^3+^, solubility and thus absorption of lomefloxacin, decresed as a result of formation of species with low solubility [143]. The permeability through intestinal mucosa of fluoroquinolone alone and in the presence of metal ions was studied *in vitro*. The effect of Ca^2+^, Mg^2+^, Fe^2+^ was tested with ciprofloxacin, while the effect of Al^3+^ was tested with ciprofloxacin, norfloxacin and ofloxacin. The experimental data revealed that the fluoroquinolone-metal ion combinations resulted in a reduced intestinal permeability compared to that of the corresponding fluoroquinolone, leading to a reduction of fluoroquinolone bioavailability [144].

### 4.3. Mechanism of Action of Quinolones

The DNA-binding capacity of quinolone complexes was studied in relation with the mechanism of action of quinolones. Experimental data suggested an interaction of quinolone-Mg^2+^ complex with DNA and gyrase and not a direct interaction of free quinolones with DNA, and a model for the ternary complex was proposed. In this model, Mg^2+^ acts as a bridge between the phosphate groups of the nucleic acid and the carbonyl and carboxyl moieties of norfloxacin, with additional stabilization arising from stacking interactions between the condensed rings of the drug and DNA bases [145]. 

Interaction of an oligonucleotide duplex and ciprofloxacin in the absence and in the presence of Mg^2+^ was studied and a model of the ternary Cf–Mg^2+^–duplex adduct orientation was proposed. Docking carried out on this model sustained the orientation of the CFX–Mg^2+^ in the minor groove of DNA [146]. 

Interaction with calf thymus DNA was investigated *in vitro* using different associations between quinolone and divalent metal ions: norfloxacin-Cu^2+^ [147], ciprofloxacin-Mg^2+^, -Cu^2+^ [148,149], levofloxacin-Cu^2+^ [150], gatifloxacin- Mg^2+^, - Cu^2+^ [149,151], -Co^2+^, -Cd^2+^ [151], fleroxacin- Mg^2+^, -Cu^2+^ [146], sparfloxacin-Mg^2+^ [149,152], -Cu^2+^ [149], -Cd^2+^ [152], -Cr(III), -Cr(VI) [153], pazufloxacin-Cu^2+^ [154].

From the experimental results, it was concluded that the metal ion plays an intermediary role in the interaction between quinolone and DNA, and the metal complex of quinolone can interact with DNA by an intercalative binding model [155,156]. *In vitro* experiments demonstrated the hypothesis that, on the one hand, DNA gyrase cannot bind quinolones in the absence of DNA, and on the other hand, the quinolone-gyrase-DNA complex is formed in the presence of Mg^2+^.

Magnesium and related metal ions affect the stability and function of topoisomerases: they reduce the stability of protein thus increasing the structural flexibility required for the structural changes involved in catalytic cycle [157,158]. On the other side, the divalent metal ions (especially Mg^2+^) might play a role in enzyme poisoning due to their ability to bind the topoisomerase II-directed drugs, including quinolones [158]. The coordination environment proposed for Mg^2+^ bound to topoisomerase IV consists in two C3/C4 oxygen atoms from a quinolone molecule chelated and four water molecules. Two of these water molecules are involved in hydrogen bonds with serine side chain hydroxyl group and with serine glutamic acid side chain carboxyl group. It was suggested that the interaction between quinolone and topoisomerases is mediated by this water-metal ion “bridge” [159]. Mutations of one of both amino acid residues that disrupt the bridge function partially or total, and thus the protein-quinolone interaction, are the most common causes of quinolone resistance [160].

### 4.4. Metal Complexes with Biological Activity

#### 4.4.1. Antimicrobial Activity

The consequence of interaction with metal ions on the biological activity of quinolones was approached in the first instance as a negative phenomenon, and some evidences of reduction in the antimicrobial activity of quinolones in the presence of metal ions [161,162] support this assumption. Two possible mechanisms were proposed for explaining the reduction of ciprofloxacin activity by metal cations. First of these, especially valid for chelates with 1:1 stoichiometry, could be a decreased permeation of the antibiotic into bacterial cells, while the second one is the formation of an inactive chelate [25]. 

However, for many chelates of quinolones obtained in solid state, an equal or superior activity was observed compared to that of parent drugs. Selected results expressed as minimal inhibitory concentration (MIC, μg mL^−1^) or as the inhibition diameter zone (mm) are presented in Table 7 and Table 8. Increased biological activity of metal chelates was explained by the overtone concept of cell permeability and chelation theory. Upon chelation, the polarity of a metal ion is reduced due to the partial sharing of positive charge with the donor groups of ligand and as a consequence of overlap with the ligand orbitals. Chelation increases the delocalization of π electrons over the whole chelate ring and thus increases the lipophilic nature of the central ion. This increased in lipophilicity enhances the passage of complex through the lipid membranes and the penetration in cells [163,164,165].

**Table 7:** Minimal inhibitory concentration (MIC, μg mL^−1^) of the drugs for some assayed bacteria.

Compound	Bacterial strain							Ref
	Gram (+)			Gram (-)				
	*S.* *aureus*	*B.* *subtilis*	*E.* *faecalis*	*E. coli*	*P. aeruginosa*	*K.* *pneumoniae*	*S.* *typhimurium*	
Pipemidic acid	16.0	-	-	64.0	64.0	-	-	[29]
[Cu(PPA)_2_(H_2_O)]	16.0	-	-	8.0	8.0	-	-	
[VO(PPA)_2_(H_2_O)]	16.0	-	-	64.0	64.0	-	-	[28]
[Mn(PPA)_2_(H_2_O)_2_]	16.0	-	-	64.0	64.0	-	-	
[Fe(PPA)_3_]	32.0	-	-	64.0	64.0	-	-	
[Co(PPA)_2_(H_2_O)_2_]	32.0	-	-	64.0	64.0	-	-	
[Ni(PPA)_2_(H_2_O)_2_]	32.0	-	-	64.0	32.0	-	-	
[Zn(PPA)_2_(H_2_O)_2_]	32.0	-	-	32.0	32.0	-	-	
[MoO_2_(PPA)_2_]	16.0	-	-	64.0	64.0	-	-	
[Cd(PPA)_2_(H_2_O)_2_]	16.0	-	-	64.0	64.0	-	-	
[UO_2_(PPA)_2_]	8.0	-	-	8.0	8.0	-	-	
Cinoxacin	> 64	-	> 64	4.0	> 64	8.0	4.0	[33]
[Cu(Cx)_2_]·2H _2_O	> 64	-	> 64	4.0	> 64	8.0	4.0	
[Co(Cx)_3_]Na·10H_2_O	> 64	-	> 64	2.0	> 64	2.0*	2.0	
Cu(Cx)(HCx)Cl·2H_2_O	> 64	-	> 64	4.0	> 64	8.0*	8.0	
[Zn(Cx)_2_]·4H_2_O	> 64	-	> 64	4.0	> 64	4.0*	4.0	
Cd(Cx)Cl·H_2_O	> 64	-	64	4.0	> 64	8.0*	8.0	
[Cd_2_(Cx)_4_(DMSO)_2_]·2H_2_O	> 64	-	64	8.0	> 64	8.0*	8.0	
[Cd_2_(Cx)_4_(H_2_O)_2_]·10H_2_O	> 64	-	64	4.0	> 64	4.0*	4.0	
Oxolinic acid	16	-	-	1	16	-	-	[35]
[Cu(oxo)_2_(H_2_O)]	64	-	-	64	32	-	-	
Enoxacin	1	0.25	4	0.12	0.12	0.12	0.12	[44]
[Co(HEx)_2_(ClO_4_)_2_]·3H_2_O	2	0.5	8	0.25	0.25	0.25	0.12	
[Co(HEx)_2_(NO_3_)_2_]·2H_2_O	1	0.25	8	0.25	0.25	0.25	0.12	
Norfloxacin	0.060	-	-	0.050	-	0.075	-	[49]
[Bi(C_16_H_18_FN_3_O_3_)_4_(H_2_O)_2_]	0.045	-	-	0.025	-	0.060	-	
Ciprofloxacin	1	0.12	1	0.03	0.5	0.03	0.016	[44]
[Cu(HCf)_2_(NO_3_)_2_]·6H_2_O	0.5	0.12	0.5	0.03	1	0.06	0.03	
[Cu(HCf)(C_2_O_4_)]·2H_2_O	0.5	0.12	2	0.06	1	0.06	0.06	
Ciprofloxacin	0.25	0.03	1	0.016	0.12	0.03	0.016	[34]
[Co(Cf)_2_(H_2_O)]·9H_2_O	0.25	0.06	1	0.004	0.12	0.016	0.008	
[Zn(Cf)_2_(H_2_O)_2_]·8H_2_O	0.25	0.03	1	0.004	0.12	0.03	0.016	
Ni(Cf)_2_· 10H_2_O	0.5	0.03	1	0.12	0.12	0.03	0.016	
Cu(Cf)_2_· 6H_2_O	0.25	0.03	1	0.004	0.12	0.03	0.008	
Ofloxacin	0.75 **	0.5	10	0.2	7	0.7	0.75 ***	[83]
[Mg(R-oflo)(S-oflo)(H_2_O)_2_]·2H_2_O	1 **	0.8	15	0.25	10	1	1 ***	
Levofloxacin	0.3 **	0.3	4	0.15	3	0.25	0.5 ***	
[Mg(S-oflo)_2_(H_2_O)_2_]·2H_2_O	0.6 **	0.5	4	0.15	5	0.5	0.75 ***	
Enrofloxacin	8	-	-	1	1	-	-	[93]
[Cu(erx)_2_(H_2_O)	32	-	-	0.125	0.125	-	-	
erx	0.012	-	-	-	-	-	-	[92]
[Cu(erx)_2_]Cl	0.0085	-	-	-	-	-	-	
Herx	8	-	-	1	1	-	-	[89]
[VO(erx)_2_(H_2_O)]	8	-	-	4	4	-	-	
[Cu(erx)_2_(H_2_O)]	4	-	-	0.125	0.125	-	-	
[MO_2_(erx)_2_]	4	-	-	1	1	-	-	

Abbreviations: *S. aureus, Staphylococcus aureus; B. subtilis, Bacillus subtilis; E. faecalis, Enterococcus (Streptococcus) faecalis; E. coli, Escherichia coli; P. aeruginosa, Pseudomonas aeruginosa; K. Pneumoniae, Klebsiella pneumoniae; S. thyphimurium, Salmonella typhimurium;* * Klebsiella spp; ** S. epidermidis; *** *S. enteriditis.*

**Table 8:** The inhibition diameter zone values (mm) for norfloxacin and some of its complexes.

Compound	Bacterial strain			Reference
	*Staphylococcus aureus*	*Escherichia coli*	*Pseudomonas aeruginosa*	
Norfloxacin	12	25	13	[63]
[Y(NOR)_2_(H_2_O)_2_]Cl_3_∙10H_2_O	31	39	47	
[Pd(NOR)_2_]Cl_2_∙3H_2_O	27	26	28	
[La(nor)_3_]∙3H_2_O	12	10	9	[64]
[Ce(nor)_3_]∙3H_2_O	12	11	10	

In fact, many more factors should be considered for metal complexes with antimicrobial activity: (i) the nature of the metal ion; (ii) the nature of the ligands; (iii) the chelate effect; (iv) the total charge of the complex; (iv) the nature of the ion neutralizing the ionic complex; and (vi) the nuclearity of the metal center in the complex [28,29,89,90,91,107]. A detailed comment of the effect of these factors on the biological activity of metal-quinolone complexes was made in a recent review [107].

The results obtained in some particular bacterial strains (*Mycobacterium tuberculosis* and *Helycobacter pylori*), which have not been included in Table 7 and Table 8, are worth emphasizing distinctively. Fluoroquinolones have been used successfully in helping cure multidrug-resistant tuberculosis, and studies in mice suggest that they can be considered as first line drugs to shorten the duration of therapy [166]. The main drawback with these agents is the high level of resistance, mainly associated with mutation at gyrA or gyrB genes [167,168]. Metal coordination to quinolones can be used not only as strategy to enhance their activity, but also to overcome the drug resistance. The complex of Cu(II) with ciprofloxacin having general formula [Cu(Cf)_2_(BF_4_)_2_]**·**6H_2_O exhibited a significant enhancement in the antitubercular activity comparing to ciprofloxacin alone [169]. A series of Pd(II) and Pt(II) complexes with general formula [MCl_2_(L)] (where L = ciprofloxacin, levofloxacin, ofloxacin, sparfloxacin, and gatifloxacin) were evaluated against *Mycobacterium tuberculosis* virulent strain H37Rv. The Pd(II) and Pt(II) complexes with sparfloxacin and the Pt(II) complex with gatifloxacin were the most active within each series in inhibiting bacterial growth, while the least active complexes of the series were the Pd(II) complex with ciprofloxacin and the Pt(II) complex with ofloxacin. Complexes have not shown better antitubercular activity than free gatifloxacin, but their activity was good and, except the complex of Pd(II) with ciprofloxacin, all of them were more active than rifampicin [79]. The results are in agreement with the *in vitro* activities of the parent drugs against *M. tuberculosis* isolated: ciprofloxacin < or = ofloxacin < sparfloxacin < gatifloxacin [170].

Fluoroquinolones from new generations, like levofloxacin, moxifloxacin, gatifloxacin or sitafloxacin have demonstrated efficacy in *Helicobacter pylori* eradication, in third-line or second-line triple therapy, in combination with a proton pump inhibitor (PPI) and amoxicilin [171,172]. Bismuth-containing quadruple therapy (omeprazole, bismuth, metronidazole and tetracycline) is an alternative first choice treatment for *H. pylori* [173]. Good results were also obtained with quadruple therapy of bismuth subcytrate-moxifloxacine-tetracycline-lansoprazole (BMTL) with high eradication rate and relatively mild side effects [174]. Starting from these premises, a series of bismuth-fluoroquinolone complexes [Bi(Flq)_3_(H_2_O)_2_] (Flq: norfloxacin, ofloxacin, ciprofloxacin, sparfloxacin, lomefloxacin, pefloxacin, gatifloxacin) were evaluated for their anti-*H. pylori* activity, and were found to be more potent against all strains of *H. pylori* used, comparing to the parent FLQs. Moreover, the synthesized complexes also showed high potency against some fluoroquinolone-resistant strains of *H. pylori.* [50]. 

#### 4.4.2. Antifungal and Antiparasitic Activity

Altough quinolones themselves does not exhibit antifungal activity some of complexes generated by newer fluoroquinolones act not only as antimicrobial agents, but have also shown antifungal activity. Complexes with 1:1 stoichiometry of levofloxacin with Cr(III), Fe(III), Co(II), Ni(II), Cu(II), Th(IV), Mn(II), Zn(II) and UO2(II) have proved an antifungal effect higher than the free ligand against Candida albicans [82]. Complexes of gatifloxacin with Ni(II), Cu(II), Zn(II), Cd(II), Fe(III), Ca(II), Mg(II),Cr(III), Mn(II) and Co(II) having a stoichiometry 1:2 (metal: ligand) have excellent activity as compared to standard drug toward the fungi *Trichophyton rubrum*, *Candida albicans* and *Fusarium solani* [100]. 

The complexes [MnCl_2_(sf)(H_2_O)_2_] and [CoCl_2_(sf)(H_2_O)_2_] displayed a considerable antiparasitic activity against *Trypanosoma cruzi*. The corresponding complexes of norfloxacin have a differentiated activity: the Mn(II) complex did not improve the anti-parasitic effect of the free norfloxacin, while the Co(II) complex displayed a 4-fold higher activity than norfloxacin ligand [53].

A new field of research was opened starting to the synthesis of organometallic ruthenium complexes of some quinolone antibacterial agents. The organometallic ruthenium complex of ofloxacin [(η^6^-p-cymene)RuCl(O,O-oflo)]·2.8H_2_O has a “piano-stool” structure with quinolone acting as bidentate ligand coordinated to the metal through the ring carbonyl and one of the carboxylic oxygen atoms [175]. The complex interacts with DNA and provokes DNA shrinkage. It is moderately active against *Trypanosoma brucei rhodesiense*, *Trypanosoma cruzi* and *Plasmodium falciparum*.

#### 4.4.3. Anticancer Activity

The anticancer activity of fluoroquinolones has been explored in the last years [176,177,178,179] based on their ability to block topoisomerase II, thus inhibiting the DNA repair activity. It is not surprising that numerous studies concerning the biological activity of quinolone metal complexes include their ability to interact with DNA, as a premise for anticancer activity (see Table 4).

Some complexes of lomefloxacin, [Co(LFX)(H_2_O)_4_]∙Cl_2_ and [Zn(LFX)(H_2_O)_4_]∙Cl_2_ were found to be very active against the breast cancer cell line MCF7 [82]. The anti-proliferative activities of the complex [Cu(mox)(H_2_O)_2_Cl]BF_4_ and of other congeneric complexes with mixed ligands were evaluated against four breast cancer cell lines (MCF-7, T47D, MDA-MB-231 and BT-20), along with the normal breast epithelial MCF-10A cell line, comparing to the parent drug, moxifloxacin. Both the parent ligand as well as its copper complex did not significantly inhibit the proliferation of non-tumorogenic MCF-10A breast epithelial cells. Moxifloxacin did not exhibit anti-proliferative effect against any of the breast cancer cell lines examined, instead, the Co(II) complexes showed differential anti-proliferative activity against the tested breast cancer cell lines [103].

The gold (III) complexes with general formula [AuCl_2_L]Cl (L = norfloxacin, levofloxacin, sparfloxacin) were tested against A20 (murine lymphoma), B16-F10 (murine melanoma) and K562 (human myeloid leukemia) tumor cell lines comparing to the normal cell lines L919 (murine lung fibroblasts) and MCR-5 (human lung fibroblasts). The free ligands did not showed significant activity in the tumor or normal cell lines, whereas the complexes are more active than the parent drugs, and they have with a similar cytotoxic activity [62]. 

Recent research has focused on increasing the antitumor activity of polyoxometalates (POMs) by introduction of medicine molecules into the POM surface [180], and such molecules could be quinolone chelates. The first compound obtained by modifying the surface of a POM with a quinolone chelate was the complex {[Co(PPA)_2_]H_2_[SiW_12_O_40_]}∙HPPA∙3H_2_O. The inhibitory effect against MCF-7 cells lines showed that the complex and pipemidic acid have shown high antitumor activity to MCF-7, whereas the parent compound SiW_12_ exhibits no antitumor activity to MCF-7. Furthermore, the antitumor activity of complex was higher that that of its parent compounds, and this superiority could be explained from the synergism of POMs and Co-PPA [113]. Other complexes of pipemidic acid, [Cu(PPA)_2_]_2_∙[PW_12_O_40_]∙6H_2_O), [HPPA]_5_∙[PW_11_CdO_39_]∙2H_2_O, and [HPPA]_3_∙[PW_12_O_40_]∙2H_2_O showed a stronger antitumor activity than that of the parent anion against PC-3, Hela and HepG2 cells [114]. 

It was found that antitumor activity depends on the binding mode of the polyoxoanion. Thus, the complex {[Ni(PPA)_2_]H_4_[SiW_12_O_40_]}∙HPPA∙3H_2_O, with a SiW_12_ polyoxoanion acting as a mono-dentate inorganic ligand covalently linked to the nickel ions, showed no antitumor activity, whereas {[Zn(PPA)_2_]_2_H_4_[SiW_12_O_40_]}∙3H_2_O, with a SiW_12_ polyoxoanion acting as a bi-dentate inorganic ligand covalently linked to the two zinc ions, exhibited higher antitumor activities than its parent compound against MCF-7 lines [115]. The type of polyoxoanion also affects the antitumor activity. This effect was observed for complexes [Cu^II^(L^1^)_2_(H_2_O)_2_]H_2_[β-Mo_8_O_26_]∙4H_2_O (1), [Cu^II^_2_(L^2^)_4_][δ-Mo_8_O_26_]∙4H_2_O (2), [Cu^II^_2_(L^3^)_2_(H_2_O)_2_][β-Mo_8_O_26_] (3), [Cu^II^_2_(L^4^)_2_(H_2_O)_4_][β-Mo_8_O_26_]∙2H_2_O (4) (where L^1^ = enrofloxacin; L^2^ = pipemidic Acid; L^3^ = norfloxacin; L^4^ = enoxacin). The complexes 1, 3, and 4 exhibited a higher effect against SGC7901 lines comparing to the parent compound, while compound 2 showed no anti-SGC7901 activity [111]. 

The organometallic ruthenium complexes chlorido(η^6^-*p*-cymene)(nalidixicato-κ^2^*O*,*O*)ruthenium(II) and chlorido(η^6^-*p*-cymene)(cinoxacinato-κ^2^*O*,*O*)ruthenium(II) were investigated as anticancer agents in human A549 (nonsmall cell lung carcinoma), CH1 (ovarian carcinoma), and SW480 (colon carcinoma) cells by means of the colorimetric MTT assay and compared to the tumor-inhibiting properties of the respective ligands. Even though the compounds were shown to be mostly non-cytotoxic to the various cell lines, the complexes and all the ligands are inactive in the three cell lines [181].

### 4.5. Analytical Applications

#### 4.5.1. Determination of Quinolones Based on Complexation with Metal Ions

**Figure 16:**
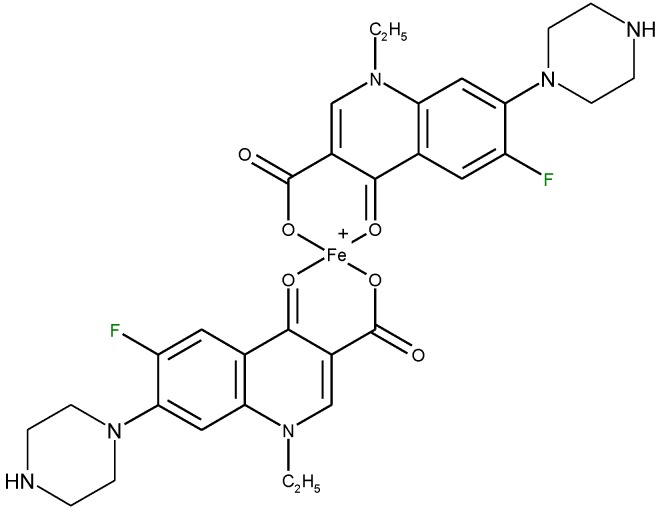
Structure of a 1:2 (metal: ligand) chelate of norfloxacin with Fe^3+^.

The capacity to form complexes with different metal ions has been applied in the analysis of quinolones in pharmaceutical formulations or in biological samples through spectrophotometric, spectroflurimetric and atomic absorption spectrometric methods. Most of the spectrophotometric methods developed for analysis of quinolones are based on the formation of yellow or orange-yellow chelates with Fe^3+^ in acid medium. The structure of such a complex is depicted in Figure 16. Generally, these methods are simple, rapid, efficient and inexpensive.

Ciprofloxacin, ofloxacin and norfloxacin have been determined colorimetrically in tablets based on their amber coloured complex with Fe(III) that exhibited a maximum at 370 nm [182]. The complex with Fe^3+^ showing a maximum absorption of 435 nm allowed spectrophotometric determination of ciprofloxacin in tablets and in solution for infusion [183]. Based on the complexation with Fe^3+^, some flow injection (FI) spectrophotometric methods for determination of norfloxacin in drug formulations were developed. The coloured Fe(III) complexes absorb at 430 nm [184] or 440 nm [185]. Ofloxacin has been also determined by a flow-injection spectrophotometric method by measuring the absorbance of its complex with Fe^3+^ at 420 nm. The method was applied for analysis of ofloxacin in pharmaceuticals and human urine [186]. Ciprofloxacin formed with Fe(III) a brown-red complex whose absorbance was monitored at 447 nm, and the developed method was used for determination of ciprofloxacin in drug formulations [187]. A sequential injection spectrophotometric method was developed for analysis of ciprofloxacin and norfloxacin by measuring the absorbance of the corresponding complexes at 447 nm and 430 nm, respectively [188]. 

Chelates with Fe(II) and Cu(II) with maximum absorptions placed below 400 nm were also applied in the spectrophotometric analysis of quinolones. Based on the yellow-coloured chelate with Fe(II) with absorbance at 358 nm, norfloxacin has been determined both in pure form and in tablet form [189]. Norfloxacin, ciprofloxacin and sparfloxacin have been determined in formulations and spiked biological fluids (plasma and urine) via their Cu(II) complexes [190]. 

Coloured ion-association complexes were applied in developing the new visible spectrophotometric methods for determination of quinolones. Ciprofloxacin and norfloxacin have been determined in pharmaceutical tablets via formation of a ternary complex with eosin and palladium (II) which showed an absorption maximum at 545 nm [191]. Ofloxacin generates with Al(III) and erythrosin an ion-association complex between {Al^III^(OFX)} cation and (ERY) anion. The ternary complex has an effective molar absorptivity at 555 nm, allowed spectrophotometrically determination of ofloxacin and other quinolone antibiotics (norfloxacin, enoxacin and levofloxacin) in pharmaceutical preparation [192]. Ion-association complexes formed with [Cr(NCS)_4_(NH_3_)_2_]^-^ (Reineckate anion) displaying a maximum absorption at 524 nm were used for determination of ofloxacin [193] and norfloxacin [194].

A spectrophotometric method related to the interaction of quinolones with metal ions was developed based on the oxidation of quinolones with ammonium vanadate in sulphuric acid medium, followed by the development of a greenish blue colour measured at 766 nm, which has been attributed to vanadium(IV). The method was applied for determination of amifloxacin, ciprofloxacin, difloxacin, enoxacin, enrofloxacin, lomefloxacin, levofloxacin, norfloxacin, ofloxacin and pefloxacin in pharmaceutical dosage forms [195].

Modification of the fluorescent properties of quinolones in the presence of different metal ions has attracted the interest for studying the interaction of quinolones with antacids [196] and for development of spectrofluorimetric methods, applied in determination of quinolones in bulk, in biological fluids and in pharmaceutical formulations.

Determination of quinolones by spectrofluorimetric methods is based on: (i) the enhancement of quinolone fluorescence in the presence of metal ions (*i.e.*, Al^3+^, Cu^2+^, Au^3+^ *etc*.); (ii) fluorescence sensitization of Tb^3+^ or Eu^3+^ in the presence of quinolone or (iii) quenching the fluorescence of a Tb^3+^ chelate after the addition of quinolone.

Interaction of a series of quinolones (sparfloxacin, oxolinic acid, flumequine and enrofloxacin) with Al^3+^ was used to analyse them in pharmaceutical dosage forms or in biological fluids [197]. Norfloxacin has been also determined as its fluorescent complex with Al^3+^ in serum [198] and in pharmaceutical preparations [199]. 

Formation of Y(III) fluorescent complexes underlying spectrofluorimetric methods for determination of norfloxacin in eye drops [200] and enrofloxacin in pharmaceutical formulations and its residue in milk [201].

The enhancement of luminescent properties of Tb(III) sorbates with ciprofloxacin and norfloxacin in zeolite was used for determination of these quinolones in biological fluids [202]. The enhancement effect of some quinolones on the fluorescence intensity of Tb(III)-sodium dodecylbenzenesulfonate system allowed the determination of enoxacin in pharmaceutical samples [203] and danofloxacin in milk [204]. Based on the sensitized fluorescence of Tb(III) enhanced by silver nanoparticles ciprofloxacin was dosed in pharmaceutical formulations [205], whereas pipemidic acid and lomefloxacin have been determined in pharmaceutical forms, urine and serum samples [206]. Europium (III)-sensitized fluorescence in the presence of quinolones was also applied for determination of quinolones ciprofloxacin, norfloxacin and gatifloxacin in pharmaceutical and serum samples [207] as for determination of ulifloxacin, the active metabolite of prulifloxacin in human serum and urine [208]. An optical sensor using Tb(III) and Eu(III) was constructed for analysis of norfloxacin and gatifloxacin in pharmaceutical and serum samples [209]. 

Quencing the fluorescence of an Eu(III)-β-diketone complex in micellar solution after the addition of pefloxacin underlying a time-resolved fluorimetric method for determination of pefloxacin in serum [210].

Apart from the main analytical applications in determination of quinolones in pharmaceutical forms and in biological samples, the fluorescent complexes were used also for other purposes. In this regard, fluorescence studies of Au(III)-norfloxacin system were carried out in order to study the association of Au^3+^ ions with cationic, zwitterionic and anionic forms of the drug [211]. Cu(II)-ofloxacin interaction, studied by means of ofloxacin fluorescence quenching experiments in the presence of Cu(II), was evaluated for its environmental impact [212].

Forming the metal complexes was the basis of some indirect methods for analysis of quinolones using atomic absorption spectrometry (AAS). Flow injection-fast atomic absorption spectroscopy (FI-AAS) was applied for determination of norfloxacin based on the complexation reaction with Fe(III), via measuring the absorbance of Fe^3+^ [213]. The formation of ion associated in the presence of cobalt sulphate was used for AAS determination of some fluoroquinolones in pharmaceutical dosage forms and biological fluids [195]. Ion-pair complexes formed with Reineckate anion allowed AAS determination of gatifloxacin, moxifloxacin and sparfloxacin in pharmaceutical formulations [214].

#### 4.5.2. Determination of Metal Ions Based on Complexation with Quinolones

Spectrophotometric and spectroflurimetric methods were developed for determination of metal ions based on their complexation with quinolones. Formation of a coloured chelate with norfloxacin, which exhibits an absorption maximum at 377 nm, was used for development of a spectophotometric method for determination of trace amounts of Fe(III) [215].

Norfloxacin was used as reagent for determination of neodymium, holmium and erbium in mixed rare earth through a derivative spectrophotometric method, based on the enhancement of absorption at 575 nm for neodymium, 450 nm for holmium, and 523 for erbium, respectively [216].

The complex between europium(III) and gatifloxacin in a co-luminiscence system Eu^3+^-La^3+^-gatifloxacin-sodium dodecylbenzene sulfonate was used for the determination of trace amounts of Eu^3+^ in rare earth samples [217]. Quencing fluorescence of a terbium chelates in the presence of Hg^2+^ was used for development of a highly sensitive and specific detection method of trace Hg^2+^ in trace Hg^2+^ in biological samples (urine) and environmental water [218]. 

#### 4.5.3. Quinolone Metal Complexes as Labels or Probes for Various Purposes

The luminescent properties of Tb(III) and Eu(III) chelates of some quinolones (nalidixic acid, oxolinic acid, pipemidic acid, pefloxacin, norfloxacin, ofloxacin, ciprofloxacin and lomefloxacin) were characterized and the obtained reagents were proposed as labels for immunofluorimetric assay [219]. Based on the enhancement of the fluorescence intensity of the enoxacin-Tb^3^^+^ complex, an environmentally friendly probe for determination of DNA (both single-stranded and double-stranded) was developed [220].

## 5. Conclusions

The metal ion - quinolone complexation represents a research field of increasing progress, having in view the consequences and applications of this process. Pharmaceutical profiles of quinolones can be improved by obtaining complexes with enhanced solubility. On the other side, pharmacokinetic interactions can occur at oral co-administration of quinolones and metal ions from mineral supplements and antacids. At the target site of their action, a quinolone-gyrase-DNA complex is formed in the presence of Mg^2+^ ions. 

Many metal ion—quinolone complexes obtained in the solid state have shown various biological effects: antimicrobial activity (sometimes equal or better than that of the parent quinolone), anticancer activity, and, in some cases, antifungal and antiparasitic activity.

Complexation with metal ions was harnessed in the development of spectrophotometric, spectroflurimetric and atomic absorbtion spectrometric methods for the determination of quinolones in pharmaceutical preparations or in biological samples. Conversely, trace metal ions can be determined using quinolones as complexing agents. It must be noted that the progresses in the field of quinolone complexes and their applications parallel the development of the newer fluoroquinolones with enlarged biological activity.
